# Letter to the Editor: Regarding Electrocardiographic Predictors of Major Adverse Cardiovascular Events in Women With INOCA

**DOI:** 10.1111/anec.70191

**Published:** 2026-04-22

**Authors:** Oguz Kaan Kaya

**Affiliations:** ^1^ Department of Cardiology Antalya Training and Research Hospital, University of Health Sciences Antalya Turkey

To the Editor,

We read with great interest the recent article by Dungan et al. (2026) evaluating electrocardiographic predictors of major adverse cardiovascular events (MACE) in women with ischemia and no obstructive coronary artery disease (INOCA). The authors should be commended for addressing an important and often underrecognized clinical entity. The use of readily available electrocardiographic (ECG) parameters to improve risk stratification in this population is particularly appealing from a practical standpoint.

However, several aspects of the study merit further consideration.

First, although resting heart rate (RHR) emerged as an independent predictor of MACE, the magnitude of its effect appears modest (hazard ratio 1.018), and the observed values largely remained within the normal physiological range. This raises the question of whether RHR represents a causal factor or rather a surrogate marker reflecting autonomic imbalance, comorbidity burden, or overall disease severity. Indeed, prior large‐scale analyses suggest that the prognostic value of RHR is often mediated through underlying risk profiles rather than direct pathophysiological mechanisms (Zhang et al. 2016).

Second, it is noteworthy that traditional ECG parameters such as QTc interval, QRS duration, and ST–T abnormalities were not independently associated with outcomes in the final model. This finding contrasts with prior evidence demonstrating the prognostic relevance of repolarization abnormalities in various cardiovascular populations (O'Neal et al. 2017), and may reflect the limited sensitivity of resting ECG in capturing the underlying pathophysiology of INOCA, which is predominantly driven by coronary microvascular dysfunction and subendocardial ischemia (Pepine 2023).

In this context, advanced imaging modalities may provide incremental value beyond ECG. Techniques such as speckle‐tracking echocardiography and global longitudinal strain (GLS) offer superior sensitivity for detecting early subclinical myocardial dysfunction, particularly at the subendocardial level (Yingchoncharoen et al. 2013). Given the central role of microvascular impairment in INOCA, integration of myocardial deformation parameters into future risk models may improve prognostic accuracy and provide a more comprehensive assessment of myocardial involvement.

Finally, although the exclusive inclusion of women is appropriate given the epidemiology of INOCA, it may limit the generalizability of the findings. In addition, the relatively high number of covariates in relation to the number of events raises the possibility of model overfitting.

In conclusion, this study provides valuable insights into the prognostic role of simple ECG parameters in INOCA. However, the modest predictive strength of RHR and the absence of other ECG markers highlight the need for more sensitive tools. Future studies integrating ECG with advanced myocardial imaging may provide a more clinically meaningful framework for risk stratification in this challenging patient population.

Sincerely,

Oguz Kaan Kaya

## Author Contributions

The author takes full responsibility for this article.

## Conflicts of Interest

The author declares no conflicts of interest.

## Data Availability

The data that support the findings of this study are available from the corresponding author upon reasonable request.

## References

[anec70191-bib-0001] Dungan, J. R. , Y. K. Taha , Q. Pei , et al. 2026. “Electrocardiographic Predictors of Major Adverse Cardiovascular Events in Women With Suspected Ischemia and no Obstructive Coronary Artery Disease.” Annals of Noninvasive Electrocardiology 31: e70168. 41902512 10.1111/anec.70168 PMC13580816

[anec70191-bib-0002] O'Neal, W. T. , M. J. Singleton , J. D. Roberts , et al. 2017. “Association Between QT‐Interval Components and Sudden Cardiac Death: The ARIC Study.” Circulation. Arrhythmia and Electrophysiology 10: e005485. 29030380 10.1161/CIRCEP.117.005485 PMC5659833

[anec70191-bib-0003] Pepine, C. J. 2023. “INOCA/ANOCA/MINOCA: Open Artery Ischemia.” American Heart Journal Plus 26: 100260. 37064505 10.1016/j.ahjo.2023.100260 PMC10104448

[anec70191-bib-0004] Yingchoncharoen, T. , S. Agarwal , Z. B. Popović , and T. H. Marwick . 2013. “Normal Ranges of Left Ventricular Strain: A Meta‐Analysis.” Journal of the American Society of Echocardiography 26: 185–191. 23218891 10.1016/j.echo.2012.10.008

[anec70191-bib-0005] Zhang, D. , X. Shen , and X. Qi . 2016. “Resting Heart Rate and All‐Cause and Cardiovascular Mortality in the General Population: A Meta‐Analysis.” Canadian Medical Association Journal 188: E53–E63. 26598376 10.1503/cmaj.150535 PMC4754196

